# From trauma‐informed approaches to self‐determination: Creating ethical spaces in health professions education research

**DOI:** 10.1111/medu.70065

**Published:** 2025-10-23

**Authors:** Lisa Richardson, Marcia Anderson

**Affiliations:** ^1^ University of Toronto Toronto Canada; ^2^ University of Manitoba Winnipeg Canada

## Abstract

Self‐determination is offered as a means to integrate trauma‐informed approaches into health professional education research without reproducing harm #trauma‐informed #self‐determination

Understanding how previous trauma can affect a person's interactions with healthcare or academic institutions has led to the integration of trauma‐informed approaches into various fields. Although trauma‐informed approaches in Health Professions Education (HPE) research are currently very limited, Nolan offers a thoughtful and concrete overview that should inspire all researchers, educators and learners to integrate trauma‐informed principles and strategies into their work.^1^ Building on ideas from Indigenous scholarship, we propose a way forward for HPE research that incorporates trauma‐informed approaches and simultaneously moves beyond them towards self‐determination.

Building on ideas from Indigenous scholarship, we propose a way forward for HPE research that incorporates trauma‐informed approaches and simultaneously moves beyond them towards self‐determination.

As Indigenous scholars in health professions education, we strongly support the integration of trauma‐informed approaches into HPE research. The experiences of Indigenous peoples and communities within healthcare, education and research spaces include egregious racism and discrimination.^2^, ^3^, ^4^ Building on ideas from Indigenous scholarship, we propose a way forward for HPE research that incorporates *trauma‐informed approaches* and simultaneously moves beyond them towards *self‐determination*.^5^ A self‐determined approach not only protects participants from the recurring trauma of existing research practices but calls on researchers, leaders and communities to imagine and create structures and systems for research that are inclusive of different ways of sharing power and of creating and disseminating knowledge. While collective self‐determination is a right enshrined in the United Nations Declaration on the Rights of Indigenous Peoples, the broader principle and practice of self‐determination offer beneficial lessons for HPE research.^6^

While understanding the above idea is a key principle for trauma‐informed research practice, it also unmasks a significant challenge: is it possible for research to be conducted by institutions or researchers affiliated with institutions which have perpetuated and continue to perpetuate racial, cultural and historical traumas without causing recurring trauma?

Is it possible for research to be conducted by institutions or researchers affiliated with institutions which have perpetuated and continue to perpetuate racial, cultural and historical traumas without causing recurring trauma?

Theorizing and enacting a space between two knowledge systems and groups of people when there are historical and ongoing power imbalances is complex. Cree legal scholar, Willie Ermine, articulated the notion of an “ethical space”.^7^ The ethical space is a framework for engagement in which societies with divergent worldviews can come together in shared dialogues of respect: *“In its finest form, the notion of an agreement to interact must always be preceded by the affirmation of human diversity created by philosophical and cultural differences.”* ^7^ In Ermine's framework, the creation of an ethical space requires historical and ongoing power imbalances to be equalized through the assertion of Indigenous rights. To create ethical spaces in HPE research, there is a need for research teams, institutions and communities to come together and work across differences. The framework for engagement, however, requires antecedent structural changes to ensure a sharing of resources and infrastructure to support research.

Self‐determination in HPE research requires the creation of an ethical space which redresses power imbalances related to resources, knowledge systems and power hierarchies (racial, cultural and level of seniority) within the institution, where communities inside and outside of the institutions can work on their own vision for HPE research. In addition to adopting strategies for trauma‐informed approaches, institutions and researchers must direct their gaze inwards to understand why and how research continues to cause harm. They must look for practices, methodologies, ideas, infrastructure and approaches that lie outside of institutions where historical and ongoing traumas occur to imagine and create ethical spaces for research in partnership with communities.

Self‐determination in HPE research requires the creation of an ethical space which redresses power imbalances.

As with trauma‐informed approaches, self‐determined research draws upon learnings from action‐based research methodologies. Furthermore, self‐determined research is strengths‐based.^8^ It identifies and builds on the strengths within an individual or community instead of studying deficits or gaps, which may pathologize the resultant trauma response rather than the systems that cause harm. A strengths‐based lens in HPE research, for example, could study the role of land‐based or experiential pedagogies in Indigenous health education rather than a deficit‐based approach that might focus on the assessment of a student's knowledge of health gaps.

The phrase self‐determination emphasizes agency and highlights an individual or community's ability to determine their needs based on their own experiences, values and knowledge systems. For example, if a local Indigenous community is interested in programs to recruit more learners into healthcare, they may choose to work with a research team to evaluate a traditional healing apprenticeship program rather than a medical school's outreach initiative. Specific strategies for an institution to enact self‐determination in research include: removing barriers so that a community member without a faculty appointment can participate in research as a principal investigator; enabling research funds to be held in a community organization; collaborating on the establishment of a community‐based research ethics board; creating robust data governance policies; offering opportunities for community members to participate as members of the research team; creating relationships for long‐term mentorship of youth to support future careers across a breadth of research; disseminating research findings with and as guided by the community. These approaches serve to create the ethical space for research outside of institutions which are active or complicit in trauma at the micro, meso and macro levels.

The phrase self‐determination emphasizes agency and highlights an individual or community's ability to determine their needs based on their own experiences, values and knowledge systems.

The integration of trauma‐informed approaches into HPE research highlights the challenge of how to reimagine research practices and structures so that trauma is not reproduced. Self‐determination offers a way forward as we consider how to create ethical spaces for generative, inclusive and rigorous health professions education and research.

## CONFLICT OF INTEREST STATEMENT

Dr. Richardson and Dr. Anderson have no conflicts of interest to declare.

## Data Availability

Data sharing not applicable to this article as no datasets were generated or analysed during the current study.
